# A critical period in the supraspinal control of pain: opioid-dependent changes in brainstem rostroventral medulla function in preadolescence

**DOI:** 10.1016/j.pain.2011.11.011

**Published:** 2012-04

**Authors:** Gareth J. Hathway, David Vega-Avelaira, Maria Fitzgerald

**Affiliations:** a UCL Neuroscience, Physiology and Pharmacology, University College London, London, UK; b School of Biomedical Sciences, The University of Nottingham, Nottingham, UK

**Keywords:** Pain, Neonate, Brainstem, Descending, Opioid, Rat, Adolescence

## Abstract

We have previously shown that the balance of electrically evoked descending brainstem control of spinal nociceptive reflexes undergoes a switch from excitation to inhibition in preadolescent rats. Here we show that the same developmental switch occurs when μ-opioid receptor agonists are microinjected into the rostroventral medulla (RVM). Microinjections of the μ-opioid receptor agonist [D-Ala_2_, N-MePhe_4_, Gly-ol]-enkephalin (DAMGO) into the RVM of lightly anaesthetised adult rats produced a dose-dependent decrease in mechanical nociceptive hindlimb reflex electromyographic activity. However, in preadolescent (postnatal day 21 [P21]) rats, the same doses of DAMGO produced reflex facilitation. RVM microinjection of δ-opioid receptor or GABA_A_ receptor agonists, on the other hand, caused reflex depression at both ages. The μ-opioid receptor-mediated descending facilitation is tonically active in naive preadolescent rats, as microinjection of the μ-opioid receptor antagonist D-Phe-Cys-Tyr-D-Trp-Orn-Thr-Pen-Thr-NH_2_ (CTOP) into the RVM at this age decreases spinal nociceptive reflexes while having no effect in adults. To test whether tonic opioid central activity is required for the preadolescent switch in RVM descending control, naloxone hydrochloride was delivered continuously from subcutaneous osmotic mini-pumps for 7-day periods, at various postnatal stages. Blockade of tonic opioidergic activity from P21 to P28, but not at earlier or later ages, prevented the normal development of descending RVM inhibitory control of spinal nociceptive reflexes. Enhancing opioidergic activity with chronic morphine over P7 to P14 accelerated this development. These results show that descending facilitation of spinal nociception in young animals is mediated by μ-opioid receptor pathways in the RVM. Furthermore, the developmental transition from RVM descending facilitation to inhibition of pain is determined by activity in central opioid networks at a critical period of periadolescence.

## Summary

The supraspinal control of spinal nociceptive processing matures during a specific period of postnatal development. This process is dependent upon the actions of endogenous opioid peptides.

## 1 Introduction

Pain modulatory networks in the brain play an active role in controlling spinal nociceptive responses so that pain perception is influenced by our state of arousal, attention and expectation. This supraspinal pain modulation is mediated by networks distributed throughout the limbic system and midbrain which exert their control at the level of the dorsal horn of the spinal cord, via anti- and pro-nociceptive descending pathways arising in the rostral ventral medulla (RVM) [4–6,10]. The importance of endogenous pain modulation via the RVM has been demonstrated in healthy human subjects and in numerous laboratory studies of models of experimental pain [8,9,11,26,44,45].

Pain experience begins in early life and noxious stimulation activates even the youngest newborn infant brain [33,34], but the importance of this early experience upon adult pain processing has been neglected in theories of pain. Responses to pain in early life are different from those seen in adults; thermal and mechanical thresholds are significantly lower in young animals and humans, and behavioural responses to noxious stimulation are uncoordinated and exaggerated [14]. More recently there is increasing evidence that children’s health and pain experience shapes their pain responses in adulthood [23,38,39]; however, the mechanism underlying this is not known.

We have previously shown that the supraspinal control of spinal nociceptive reflexes is slow to develop over the postnatal period [14,18]. Specifically, microstimulation of RVM networks is pronociceptive until postnatal day 21 (P21), after which time a clear antinociception emerges [18]. The timing of this change suggested that the process may be vulnerable to activity dependent changes in early life. A major contribution to RVM pain control arises from the central actions of endogenous opioids. In the adult, component nuclei of the pain-modulating circuit are linked through the release of endogenous opioids, which influence pain modulation by acting upon opiate receptors within the circuit, including the RVM. Thus microinjection of morphine, μ-opioid or δ-opioid receptor agonists into the adult RVM elicits analgesia by activating descending anti-nociceptive controls of dorsal horn excitability and microinjecting naloxone, an opiate receptor antagonist, in the same region is pronociceptive [12,21]. We hypothesise here that, because RVM networks are pronociceptive before P21, microinjection of μ-opioid receptor agonists into the RVM of young animals activates descending control of nociceptive reflexes, but that the effect is facilitatory.

Endogenous opioids are known to play a role in synaptogenesis and in brain development [28,43], their role in the growth and development of pain pathways is not known. We hypothesise that opioid networks have a trophic role in the maturation of supraspinal pain control and that the transition from descending facilitation to inhibition in the preadolescent period is dependent upon endogenous opioid signalling in the immature brain.

Using electromyography (EMG) recordings in response to noxious mechanical stimulation, we show that spinal excitability is differentially modulated by intra-RVM microinjection of the μ-opioid receptor agonist DAMGO in preadolescent and adult rats. Using awake behaving rats, we show that there is tonic ongoing opioid-dependent facilitation of reflexes in preadolescent but not adult rats. We further identify a critical period when this tonic endogenous opioid activity determines the normal maturation of supraspinal pain control.

## 2 Methods

All animal procedures were licensed by the UK Home Office and performed in accordance with the Animals (Scientific Procedures) Act 1986. Sprague–Dawley rats were housed in cages of 6 age-matched animals with free access to food and water. The room in which animals were housed had a 12-hour light/dark cycle. For the purposes of this study animals <40 days old are called juvenile or preadolescent, and animals ⩾40 days old are called adult. Although still only young adults, 40-day-old rats display all the structural and functional properties of mature adults, weigh between 200 and 250 g, and are routinely reported in the literature as adults.

### 2.1 RVM microinjections and electrical stimulation

Animals were anaesthetized with isoflurane and mounted on a stereotaxic frame, the skull exposed and bregma located. Stereotaxic coordinates for the RVM were calculated (adult: left–right [L-R], 0 mm; anterior–posterior [A-P], 9.7 mm; dorsal–ventral [D-V], −10 mm; P21: L-R, 0 mm; A-P, 9.2 mm; D-V, −10.0 mm) and a 30-gauge 2.5-μL syringe (Hamilton, Reno, Nevada) inserted via a drilled hole in the skull. Drugs were injected over a 5-minute period. The syringe was removed, and the wound closed.

The following drugs were administered by microinjection into the RVM at doses determined from previously published studies in adult brainstem (in parentheses): DAMGO (μ-agonist, 3, 10, and 30 ng, Sigma [22]; D[Ala]-deltorphin II, 30, 300, and 1000 ng, Tocris, Bristol U.K. [21]); gabazine (GABA_A_ receptor antagonist, 10 ng, Sigma [19]); CTOP (100 ng, Sigma [37]); and saline. Drugs were applied slowly over a 5-minute injection period, in volumes of 0.25 μL. Only 1 drug at a single dose was administered to any animal. Dose–response studies are therefore produced from separate groups of animals at each dose. In a separate group of rats, naloxone hydrochloride (2 mg/kg, i.p., Sigma (Poole U.K.)) was administered to preadolescent (P21) rats after RVM microinjection of DAMGO 300 ng 10 minutes previously and allowed to distribute for 2 minutes before remeasuring evoked reflex activity. The experimenter was blinded with regard to drug type.

For electrical stimulation, concentric bipolar stimulating electrodes were lowered into the RVM using the coordinates above. Trains of stimuli of 500-microsecond pulse width were applied at 10 Hz, at amplitudes ranging from 5 to 200 μA (Neurolog; Digitimer, Welwyn Garden City, UK).

### 2.2 EMG recording

EMG recordings were performed as described previously [18]. Animals were anesthetized with isoflurane (2%–4%) and an endotracheal cannula inserted for artificial ventilation and then mounted on a stereotaxic frame (Kopf Instruments Tujunga, California). A bipolar concentric needle electrode (Ainsworks, coventry, UK) was inserted in the lateral biceps femoris through a small skin incision. Such recording electrodes ensure that recorded activity is restricted to local muscle activity in small animals. Isoflurane anesthesia was then reduced to 1.5% (Univentor 400 anesthesia unit, Malta) and equilibrated for 20 minutes before recording. Flexion EMG (full-wave rectified) activity was recorded following sequential (lowest to highest intensity) von Frey hair (vFh) stimulation of the plantar surface of the foot. Because mechanical withdrawal thresholds are significantly lower in neonates than in adults [14], different hairs were used in each age group. Thresholds were determined as the von Frey hair that produced an EMG response that was 10% greater than resting EMG activity.

**Fig. 1:**
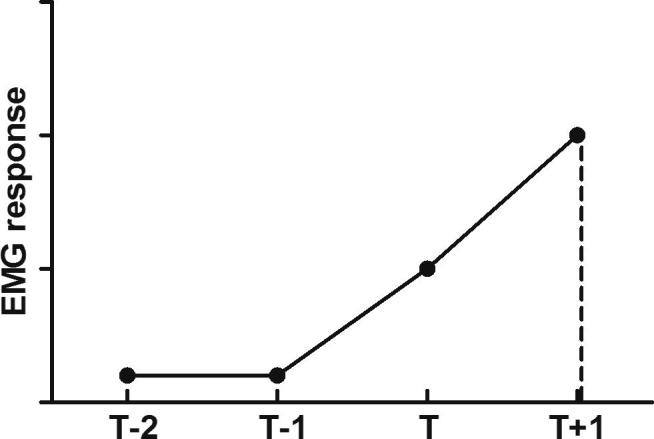
Method of determining spinal excitability using EMG recordings. Four von Frey hairs are applied sequentially to the plantar surface of 1 hindpaw, and the flexor reflex EMG is recorded. Under basal conditions, the threshold is determined (T) and the responses to this, to 1 hair above threshold (T+1) and to the 2 hairs below threshold (T−1 and T−2), are measured and plotted as a stimulus–response curve. The area under the curve (AUC) is calculated for each condition, and the result is termed ‘reflex excitability’. The EMG response to these same 4 hairs, in all experimental conditions in the same animal, are then remeasured, thus allowing changes in spinal excitability to a pre-established set of noxious and innocuous stimuli to be compared.

Responses to 2 sub-threshold von Frey hairs (T−1, T−2), the threshold hair (T) and a supra-threshold hair (T+1) were recorded and the same 4 hairs used in all subsequent stimulation conditions and for data analysis (described below). Each hair was applied 3 times, and the mean reading for each of the 3 presentations recorded. A stimulus–response curve of EMG magnitude versus mechanical stimulus intensity was plotted, and the area under the curve (AUC) was calculated to provide an integrated measure of the spinal “reflex excitability” (see Fig. 1). This value was denoted the baseline response of the animal. Responses were normalized to this baseline because of variations in background (nonevoked) EMG activity. After RVM manipulations (electrical or pharmacological), the EMG responses evoked by the same 4 hairs, were remeasured and the AUC at this time (or stimulus intensity) recalculated and compared with the baseline response. Increases in EMG activity are therefore reported as positive increases in percent EMG response, whereas inhibition of EMG activity is reported as a reduction in percent EMG response (i.e., a reduction in reflex excitability). It should be noted that although facilitation of responses could be very great (i.e., >100%), inhibition of EMG activity cannot exceed −100%, and this should be considered when comparing inhibition and facilitation in the results.

### 2.3 Naloxone-filled osmotic pump insertion

Alzet mini-osmotic pumps (Model 2001; Durect Corporation, Cuppertino, CA:) were filled with a naloxone hydrochloride or naloxone methiodide (Sigma) solution (0.3 mg/kg) to deliver at a rate of 1 μL per hour for 7 days. Pumps were implanted subcutaneously in P14, P21, or P28 rats under isoflurane anesthesia and to rest upon the lower ribcage. The incision was closed with suture and animals were returned to their home cage for 19 days, after which EMG recordings in response to RVM electrical stimulation were performed (see above). Depletion of drug from the pump reservoir was confirmed post mortem. In a separate study, mini-pumps that contained morphine sulphate (0.175 mg/kg) or saline were inserted, as described above, into P7 rat pups. Drug was delivered for 7 days and animals were allowed to mature until P21, when EMG responses to RVM electrical stimulation were recorded. In all cases, the experimenter was blinded to the drug treatment of the animal.

### 2.4 Behavioural assessment of mechanical withdrawal thresholds

Animals were handled daily for 7 days before behavioural testing to acclimate them to human contact and to habituate them to the testing procedure, thereby limiting stress-induced effects. Animals were lightly restrained by the experimenter with 1 hand, and mechanical withdrawal thresholds were measured by the application of vFh to the dorsum of the foot. Hairs of increasing weight were applied sequentially, lowest to highest, 5 times each, and the first hair to elicit a withdrawal in 2 of those 5 presentations (40%) was considered to be the threshold. After baseline threshold assessment, animals received an i.p. injection of naloxone hydrochloride (2 mg/kg), naloxone methiodide (2 mg/kg), morphine sulphate (2 mg/kg), CTOP (0.1 mg/kg), or saline. Animals were returned to a holding cage and thresholds reassessed 30 minutes after drug administration in the same way. The experimenter was blinded to the drug that was being administered.

### 2.5 Immunohistochemistry

Immunohistochemistry for the μ-opioid receptor was performed on brains from rats chronically treated with naloxone hydrochloride or saline between P21 and P28. Brainstem immunohistochemistry was performed on 40-μm frozen sections after conventional 4% paraformaldehyde (PFA) perfusion and post-fixation in 4% PFA for 1 h at 4°C followed by sucrose cryoprotection for at least 2 days. In the brainstem, mouse anti-NeuN (Chemicon, Watford, U.K. 1:1000) and rabbit anti-μ-opioid receptor antibodies (Neuromics, Edina MN 1:1000) were used with goat anti-mouse (Alexa-593) and goat anti-rabbit (Alexa-488, Invitrogen, Paisley, U.K.) secondary antibodies, 1:200. Immunohistochemically stained sections were observed with a Leica IRE2 fluorescence microscope and captured using Velocity 5.5 (Perkin Elmer, UK). Four brains per treatment group were processed in this way. For each brain, 6 coronal sections at an anatomical level that included the nucleus raphe magnus were assessed. Total numbers of μ-opioid receptor-positive cells were counted in the RVM in each section, and the mean number of cells was calculated for each animal (total cells counted in 6 sections divided by 6). These mean values in each treatment group were then compared statistically (discussed below).

### 2.6 Quantitative polymerase chain reaction

RNA was extracted from freshly dissected P21 and P40 RVM using Trizol (Sigma) as previously described [16], and the concentration was determined using the “Nanodrop ND-1000” system (Nanodrop Technologies). Samples were then diluted to an equal concentration (1 μg/μL) and a 10-μg quantity was used to synthesize cDNA using the InVitrogen Superscript First Strand Synthesis System for RT-PCR kit. Specific primers for μ-, κ-, and δ-opioid receptors were designed and synthesized by Sigma-Genosys. An SYBR green-based system (Applied Biosystems) was used for amplification and detection. The amplification process was monitored with Opticon Monitor v3.1.32 software (M.J. Genework Inc Waltham Mass), and amplification was normalized to β-actin housekeeping gene.

The quantitative polymerase chain reaction primer sequences were as follows: κ-opioid receptor primer sense SKOR1: gacttctgcttccccattaagatg and antisense AKOR1: ttattcatcccacccacatccc, derived from sequence accession number D16829; δ-opioid receptor primers sense SDOR1: atctgggtcttggcttcagg and antisense ADOR1: ccatagcacacggtgatgatg, derived from sequence accession number U00475; μ-opioid receptor primers sense SMOR1: tcaccagatattcaccctctg and antisense AMOR1: cgttgacgattttggcatttcg, derived from sequence accession number L22455; and β-actin primers sense SA1: agattactgccctggctccta and antisense AA1: aggatagagccaccaatccac, derived from sequence accession number NM-031144.

### 2.7 Data analysis and statistics

EMG data were normally distributed, and comparisons were made between the baseline value of the AUC and those obtained following RVM manipulation. Statistical comparisons between the time points, or stimulation intensities post RVM manipulation were achieved using a one-way analysis of variance (ANOVA) with Dunnet’s post-test. Comparisons of drug effects in dose–response studies were made using 2-way ANOVA with Bonferroni post-tests. Comparisons between control and drug treated groups were achieved using a Student’s *t* test. Behavioural data were not normally distributed and were analysed with a Kruskal–Wallis test with a post-hoc Dunn’s multiple comparison test. Immunohistochemical comparisons were made using an unpaired *t* test.

## 3 Results

### 3.1 μ-Opioid receptor activation in the RVM facilitates nociceptive reflexes in juvenile rats and inhibits them in adults

**Fig. 2:**
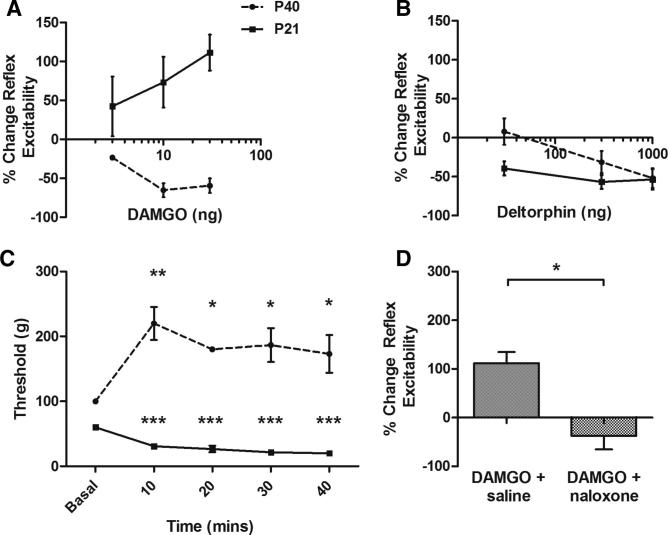
Activation of μ-opioid receptors in the RVM has differential effects on spinal excitability depending on age. (A) DAMGO microinjected into the RVM of lightly anaesthetised rats elicited dose-dependent inhibition of spinal nociceptive reflex activity in the adult rats (P40; circles) but facilitation in preadolescent rats (P21; squares). The effect of DAMGO was significantly different when the 2 age groups were compared and the inhibitory and facilitatory actions of the drug were significant at 10 and 30 ng in both age groups (*P* < 0.001 for overall response to DAMGO across doses in both age groups (F_1,16_ = 34.73). (B) Identical investigations using the δ-opioid receptor agonist D[Ala]-deltorphin II, fails to show any significant age-related difference with analgesia being observed in both groups. (C) Intra-RVM microinjection of DAMGO (30 ng) had a significant effect on the von Frey hair threshold in EMG experiments. There was a significant effect of age upon mechanical threshold (two-way ANOVA, F_1,11_ = 278.8, *P* < 0.0001). Note that the basal mechanical thresholds in the adult and preadolescent groups are different; this is consistent with previous studies showing that mechanical and thermal thresholds are lower early in life [14]. In adult rats, thresholds increased significantly from baseline (^∗∗∗^*P* < 0.001, ^∗^*P* < 0.05, Bonferroni post-test) for 30 minutes after drug microinjection. However, in preadolescent (P21) rats, thresholds decreased significantly from baseline (^∗∗∗^*P* < 0.0001, Bonferroni post-test), and this effect was maintained for at least 40 minutes after DAMGO injection. (D) The ability of 30 ng DAMGO to facilitate juvenile responses was completely reversed by s.c. naloxone hydrochloride (2 mg/kg) but was unaffected by saline [t(6) = 4.099, *P* < 0.05].

Opioid receptor activation of brainstem descending pathways inhibits spinal nociceptive circuits in adult rats [22,35]. Because descending control from the rostroventral medulla (RVM) undergoes marked functional changes in postnatal life [18], we tested whether there are differences in the effects of brainstem opioid activation upon spinal nociceptive reflexes between young and adult rats. In these experiments, nociceptive reflex excitability was measured from the EMG activity of flexor hindlimb muscles evoked by mechanical stimulation of the plantar hindpaw. As expected, microinjection of the μ-opioid receptor agonist DAMGO (3, 10, and 30 ng) into the RVM of lightly anaesthetized adult rats dose-dependently decreased spinal nociceptive reflex excitability (Fig. 2A). When the same experiment was performed in preadolescent rat pups (postnatal day 21 [P21]), the pattern of opioid receptor-mediated descending control was different; microinjection of DAMGO at the same doses facilitated rather than inhibited nociceptive reflex excitability (Fig. 2A). The enhanced reflex activity seen in preadolescent rats is not an effect of repeated testing as D[Ala]-deltorphin II, a δ-opioid receptor agonist (30, 300, and 1000 ng) did not produce age-dependent differences in excitability (Fig. 2B). Changes in reflex excitability were accompanied by a parallel change in mechanical threshold in both adults and preadolescents. In adult rats, vFh threshold significantly increased 10 minutes after DAMGO (30 ng) microinjection into the RVM (Fig. 2C). This increase was maintained for 30 minutes post-drug application. In preadolescent rats, however, a significant reduction in threshold accompanied the increase in reflex excitability. The reduction in threshold was apparent at 10 minutes after DAMGO (30 ng) and was maintained for at least 40 minutes after drug administration (Fig. 2C). To further assess whether the ability of DAMGO to facilitate preadolescent responses was due to non-μ-opioid receptor-mediated events, we tested whether the effects were reversed by opioid-receptor antagonism. Systemic application of the opioid receptor antagonist naloxone (2 mg/kg, s.c.) completely abolished the facilitatory effect of DAMGO (30 ng) on spinal nociceptive reflex excitability in young rats (Fig. 2D).

**Supplementary Fig. 1:**
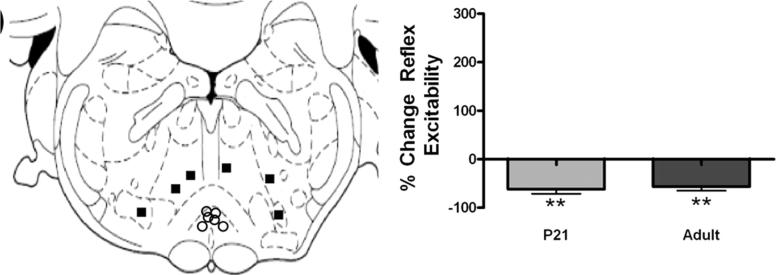
(a) Postmortem identification of RVM microinjection sites (open circles) confirmed that injections were located in or closely opposed to the nucleus raphe magnus. Control injections (filled squares) were outside the nucleus raphe magnus. (b) Microinjection of gabazine (10 ng) into the RVM of preadolescent and adult rats mediated the same effect in both age groups. Significant inhibition of spinal excitability was seen (^∗∗^*P* < 0.01, *t* test, drug vs baseline) at both ages.

To ensure that the facilitatory effect of brainstem μ-opioid receptor activation in young rats was specific to the RVM, control microinjections of DAMGO (30 ng) were made into adjacent areas of the brainstem (see Supplementary Fig. 1). These microinjections had no effect upon nociceptive reflex activity (data not shown).

To investigate whether the facilitatory effect of DAMGO was secondary to a developmental change in inhibitory neurotransmission, the GABA_A_ receptor antagonist gabazine (10 ng) was injected into the RVM of adult and preadolescent rats. In rats of both ages, gabazine inhibited spinal reflex excitability, significantly reducing evoked EMG excitability (Supplementary Fig. 1). Thus, the facilitation of spinal excitability observed upon activating immature RVM μ-opioid networks is not secondary to a shift in RVM GABA_A_ neurotransmitter systems.

Together these experiments show that activation of μ-opioid receptors in the RVM facilitates spinal nociceptive reflexes in preadolescent rats, although the same activation inhibits nociceptive reflexes in adult rats.

### 3.2 Nociceptive reflexes in preadolescence are under tonic μ-opioid receptor facilitatory control, mediated at least in part, by the RVM

Although the experiments above show that exogenous activation of RVM μ-opioid receptors facilitates nociceptive reflexes in preadolescent rats, they do not indicate whether descending excitatory control is a normal feature in young rats. If there is tonic descending excitation in naive preadolescent rats that is mediated by endogenous opioids in the brainstem, local administration of μ-opioid antagonists to the RVM should reduce the magnitude of baseline nociceptive reflexes.

**Fig. 3:**
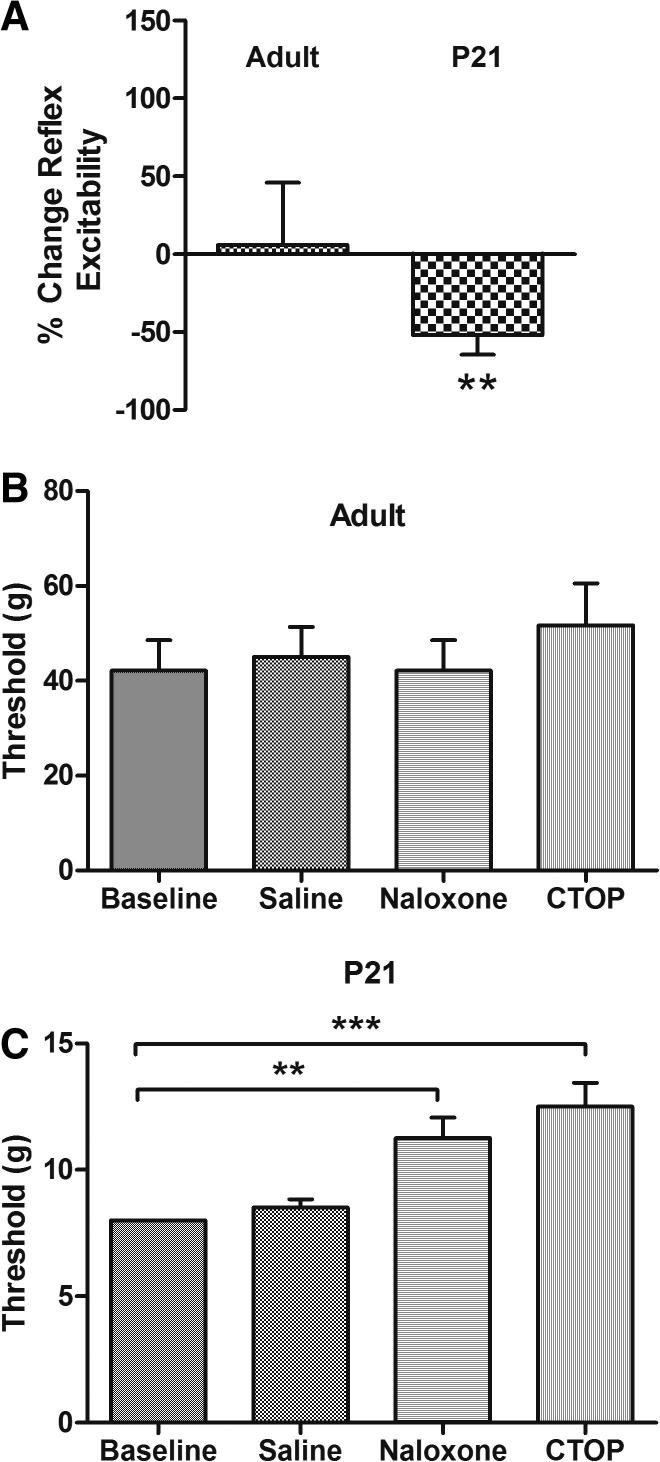
Tonic facilitation of spinal reflexes from the RVM by endogenous opioids in preadolescent rats. (A) The μ-opioid receptor antagonist CTOP (100 ng) had no effect on adult reflex activity when microinjected into the RVM of lightly anaesthetised rats, but significantly inhibited reflex magnitude in P21 animals. This shows that μ-opioid receptor-dependent facilitation arises from the pre-adolescent RVM. (B) In freely behaving adult rats, neither CTOP (0.1 mg/kg i.p.) nor naloxone hydrochloride (2 mg/kg i.p.) significantly affected mechanical thresholds. (C) The same drugs at the same doses in preadolescent rats mediated significant increases in mechanical threshold (^∗∗^*P* < 0.01, ^∗∗∗^*P* < 0.001, Dunn’s multiple comparisons test).

To test this, we microinjected the specific μ-opioid receptor antagonist CTOP (100 ng) into the RVM of lightly anaesthetised preadolescent (P21) and adult (P40) rats. Fig. 3A shows the effect of antagonizing RVM μ-opioid receptors upon the nociceptive reflex excitability in adult and P21 rat pups. Although adult responses were not affected, the response in preadolescent rats was significantly reduced (−52% ± 12%, *P* < 0.01, t(8) = 4.135 vs adult).

The presence of a tonic endogenous opioid-mediated facilitation of spinal nociception in juvenile rats was also observed upon application of systemic μ-opioid antagonists to freely behaving awake rats. Subcutaneous administration of CTOP (0.1 mg/kg) or naloxone hydrochloride (2 mg/kg, i.p.) had no effect on the mechanical threshold in adult rats (Fig. 3B). In preadolescent rats, however, both CTOP and naloxone significantly increased mechanical thresholds 30 minutes after drug administration from a baseline of 8 g to 11.25 ± 0.8 g after naloxone and 12.5 ± 0.9 g after CTOP [H(4) = 23.89, *P* < 0.001, Kruskal–Wallis].

These experiments show that the preadolescent spinal cord is under tonic descending facilitatory control, which is due at least in part to endogenous opioid activity in RVM, and that this is not present in the adult.

### 3.3 Endogenous opioid activity over a critical preadolescent period is required for the functional maturation of supraspinal RVM inhibitory pain control

The existence of a tonic μ-opioid receptor-mediated activation of the RVM that is developmentally regulated, suggests that endogenous opioids may play a trophic role in the RVM. We have previously shown that between the ages of P21 to P25, descending control of the RVM upon spinal nociceptive circuits shifts from excitation to inhibition, suggesting that, over this critical period, RVM circuitry undergoes a key maturational change [18]. The discovery of endogenous opioid activity in the preadolescent brainstem led us to hypothesise that the shift from supraspinal facilitation to inhibition of spinal nociceptive pathways at this age depends upon tonic endogenous opioid receptor activation. Specifically we proposed that the period from P21 to P25 may be a time when endogenous opioid activity has a critical trophic influence over the maturation of brainstem descending control circuits.

**Fig. 4:**
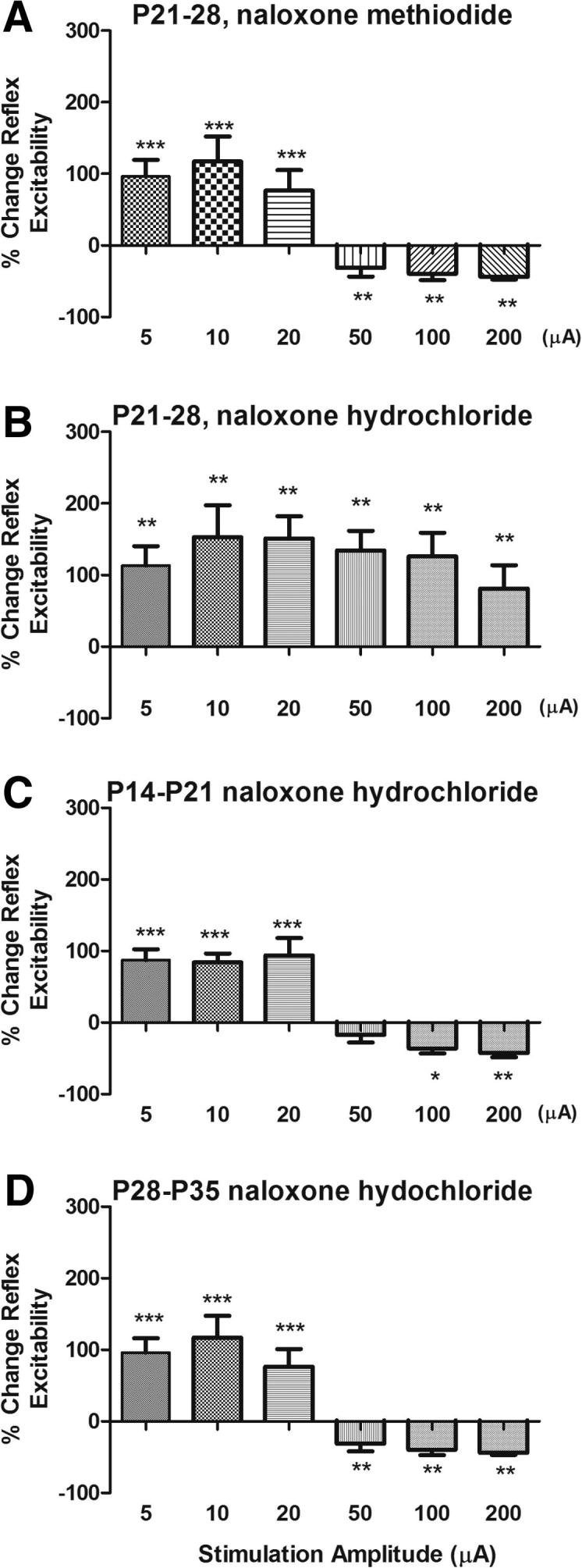
Endogenous opioid activity is required for the maturation of descending control of nociception. Bar charts showing the change in spinal nociceptive reflex excitability produced by electrical stimulation of the RVM (500 μsec, 5–200 μA, 10 Hz) in lightly anaesthetised adult rats. In each case, rats had undergone chronic administration of naloxone (3 mg/kg) via s.c. implanted osmotic mini-pumps for 7 days at different periods of adolescence. (A) Control, naloxone methiodide, which does not cross the blood–brain barrier, was administered between P21 and P28. This had no effect on the maturation of RVM descending control of nociceptive reflexes, with a significant effect of RVM stimulation compared to basal EMG responses (F_6,5_ = 17.19, *P* < 0.0001). (B) Chronic blockade of endogenous opioids from P21 to P28. Naloxone hydrochloride, which does cross the blood–brain barrier, was administered between P21 and P28. Descending inhibition, normally observed in adults at higher RVM stimulation amplitudes, has not developed (basal vs RVM stimulation, F_6,7_ = 7.456, *P* < 0.0001). (C) Chronic blockade of endogenous opioids with naloxone hydrochloride, from P14 to P21. Descending inhibition has developed as normal (basal vs RVM stimulation, F_6,5_ = 15.06, *P* < 0.0001). (D) Chronic blockade of endogenous opioids with naloxone hydrochloride, from P28 to P35 (basal vs RVM stimulation, F_6,7_ = 17.19, *P* < 0.0001). Descending inhibition has developed as normal. Bars indicate standard error of the mean. Asterisks indicate that stimulation amplitudes are significantly different from baseline in Bonferroni post-test. ^∗^*P* < 0.05, ^∗∗^*P* < 0.01, ^∗∗∗^*P* < 0.001.

To test this, rats were implanted with subcutaneous osmotic mini-pumps containing naloxone hydrochloride at P14, P21, or P28 and the drug delivered continuously for 7 days, after which time the animals were allowed to mature until adulthood (P40). Control rats were treated with chronic naloxone methiodide (which does not cross the blood–brain barrier) at P21 for 7 days. When the rats had reached adulthood, the functional status of brainstem descending control was assessed in all groups. The influence of descending RVM activation upon spinal nociceptive reflex excitability was quantified by measuring EMG responses during electrical stimulation (5–200 μA) of the RVM, as previously described [18]. Fig. 4A shows the effect of graded electrical stimulation of the RVM upon nociceptive reflex excitability in the P40 naloxone methiodide adult control group. The pattern of reflex facilitation at low RVM stimulus intensities and inhibition from 50–200 μA is exactly the same as that described for naive P40 (adult) rats [18], showing that chronic naloxone methiodide, which does not cross the blood–brain barrier, has had no effect upon the normal development of RVM descending controls.

The responses of animals treated with naloxone hydrochloride between P21 and P28 were significantly different (main effect of treatment, 2-way repeated-measures ANOVA, F_1,12_ = 7.28, *P* = 0.0194). Fig. 4B shows that chronic opioid receptor blockade over P21 to P28 led to reflexes that were significantly facilitated at all RVM stimulation intensities tested. This pattern, never observed in normal adult animals, resembles the descending control in younger P21 animals [18]; no inhibition of EMG activity by RVM stimulation was observed at any stimulation intensity. This failure of descending inhibition to mature occurred only if endogenous opioid activity was blocked over the postnatal week 4 (P21–28). Descending inhibition developed normally if rats were implanted with naloxone hydrochloride osmotic mini-pumps 1 week earlier, P14–P21 (Fig. 4C) or 1 week later (P28–P33) (Fig. 4D).

**Supplementary Fig. 2:**
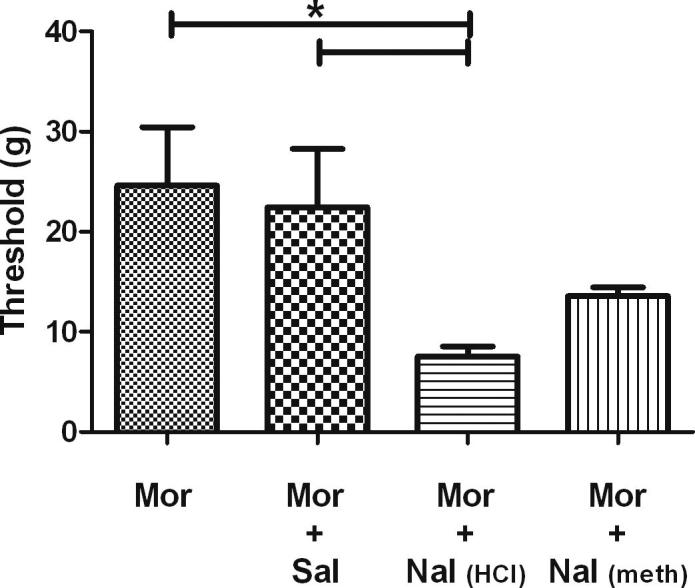
Naloxone reversal of systemic morphine does not differ in preadolescent and adult rats. (a) Morphine sulphate (2 mg/kg i.p.) produced robust analgesia in P21 rats (column Mor). Behavioural mechanical withdrawal thresholds were increased, and naloxone hydrochloride was able to significantly affect this (Kruskal–Wallis test, H(4) = 10.47, *P* = 0.015) (column Mor + Nal HCl vs column MOR, *P* < 0.05, Dunn’s multiple comparison), whereas naloxone methiodide (2 mg/kg i.p.) did not (column Mor + Nal met vs column MOR). Rats treated with morphine and saline (column Mor + Sal) were not significantly different to rats solely treated with morphine (column MOR) but did significantly differ from rats given morphine and naloxone hydrochloride (column MOR + Nal HCl ^∗^*P* < 0.05 Dunn’s multiple comparison).

**Fig. 5:**
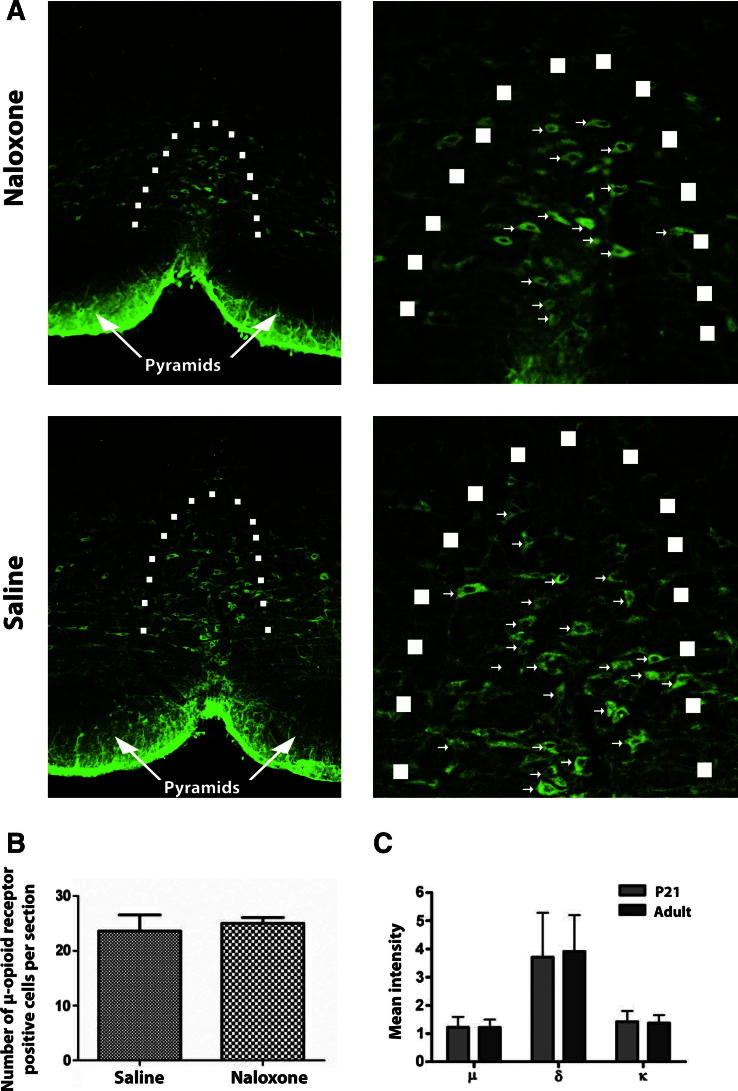
μ-Opioid receptor expression does not differ between ages or between rats chronically treated with s.c. naloxone or saline. (A) Immunohistochemical staining of the RVM from adult animals treated with naloxone hydrochloride for 7 days via osmotic mini-pumps between P21 and P28 (top) and those given chronic saline over the same period (bottom) shows that the drug treatment has no effect on μ-opioid receptor expression pattern or number of μ-opioid receptor expressing cells (arrows). Dotted line demarcates the border of the nucleus raphe magnus. (B) There are no differences in the number of μ-opioid receptor expressing neurones between the treatment groups (n = 4 per group). (C) Reverse transcription-polymerase chain reaction from rat homogenates of rat RVM at postnatal day 21 and 40 show no differences in the expression level of μ-, δ-, or κ-opioid receptors (n = 4 per group). Mean intensity was normalised to β-actin.

To confirm that systemic naloxone is a potent opioid antagonist over the developmental period of these experiments, we showed that naloxone HCL (2 mg/kg, i.p.), but not naloxone methiodide (2 mg/kg, i.p.), reversed morphine analgesia in freely behaving preadolescent rats (Supplementary Fig. 2). Furthermore, immunostaining in adult rats that had been treated with chronic naloxone between P21 and P28, revealed no difference in μ-opioid expression in the RVM compared with saline controls (Fig. 5A and B). In addition, the level of expression of μ-, δ-, and κ-opioid receptor mRNA in normal preadolescent and adult rat RVM was compared using reverse transcription-polymerase chain reaction and was found to be the same (Fig. 5C).

Together, these results show that postnatal week 4, which is the preadolescent stage in rats, represents a critical period during which endogenous activation of CNS opioid receptors is required to establish the normal balance of RVM descending inhibitory control of spinal pain circuits.

### 3.4 Chronic morphine administration in early life accelerates the maturation of RVM inhibitory pain control

**Fig. 6:**
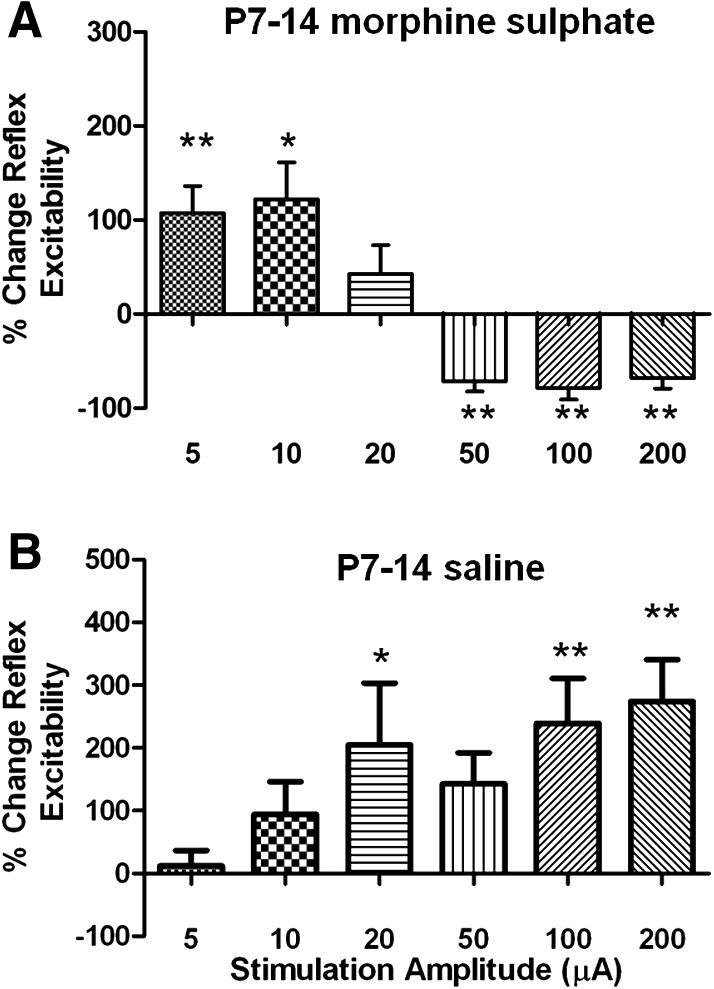
Chronic morphine application in postnatal week 2 accelerates the maturation of RVM descending control of nociception. (A) Histogram showing the change in spinal nociceptive reflex excitability produced by electrical stimulation of the RVM (500 μsec, 5–200 μA, 10 Hz) in lightly anaesthetised P21 rats that had undergone chronic administration of morphine (0.175 mg/kg) via s.c. implanted osmotic mini-pumps for 7 days between P7 and P14. In these animals, an adult pattern of descending control has developed prematurely. (B) In control saline-treated animals, facilitation was observed only at the age that is the case in normal animals of this age [18]. Bars indicate standard error of the mean. Asterisks indicate significantly different from baseline in Bonferroni post-test. ^∗^*P* < 0.05, ^∗∗^*P* < 0.01.

The results here show that endogenous activation of CNS opioid receptors during a critical period of preadolescence is required for RVM maturation. We next hypothesised that excessive activation of the same receptors by chronic administration of exogenous opioids before this critical period might therefore accelerate this maturation. To test this, we administered low doses (0.175 mg/kg) of morphine sulphate via osmotic mini-pumps for 7 days in the second postnatal week (P7–P14) and then measured the RVM descending control of spinal nociceptive reflexes 1 week later, at P21. In normal rats of this age, RVM microstimulation evokes a monophasic facilitation of nociception over the range of stimulation amplitudes tested (see Hathway et al. [18]). However, as shown in Fig. 6A, rat pups exposed to excess exogenous opiates over the second postnatal week had developed precociously, and significant descending inhibition could also be evoked at higher stimulus intensities. This pattern is normally only observed in older animals of P25 or more [18]. A control group in which animals of the same age were implanted with mini-pumps containing saline was included in this study (Fig. 6B) and there was a significant difference between these animals and those treated with morphine (2-way repeated-measures ANOVA, morphine vs saline, F_1,11_ = 7.29, *P* = 0.0223). Saline-treated animals at this age failed to exhibit any descending inhibition. These data show that the normal development of supraspinal RVM control of spinal nociceptive circuits depends on the opioid receptor-mediated activity in the brain in preadolescent life.

## 4 Discussion

The involvement of supraspinal centres in the modulation of nociception has been recognised for more than 50 years, since the work of Hagbarth and Kerr [17]. The gate control theory of pain of Melzack and Wall [27] was the first clear articulation of the central role of these descending pathways in processing noxious sensory information, and since then it has become clear that endogenous supraspinal mechanisms exert powerful control over spinal cord pain circuits mediating such phenomena as placebo analgesia [10] and playing a key role in the maintenance of chronic pain states [29]. Although there is increasing understanding of the neuronal networks and neurotransmitter systems that mediate descending facilitatory and inhibitory control of pain (for reviews, see Fields et al. [13] and Zhuo and Gebhart [46]), knowledge of how these pathways are established in early life and their functional maturation through adolescence is lacking. This is surprising when it is considered, that once hospitalised, children are just as likely to undergo painful procedures as adults, and that pain in early life can significantly alter sensory processing for a significant period extending beyond maturation and into later life [32,38,39]. In this study, we have demonstrated that opioid receptor-mediated activation of circuits in the RVM undergo significant changes in the preadolescent period of the rat. We have demonstrated that μ-opioid receptor-mediated descending activity from the immature RVM is pro-nociceptive, and furthermore that spinal nociceptive circuits are under tonic opioid receptor-mediated facilitation in preadolescence. After this time, the tonic facilitation decreases and RVM μ-opioid receptor-mediated responses become anti-nociceptive in the adult. Sufficient levels of endogenous opioids during a critical period of development are essential to this maturational process, and if opioid activity is blocked between P21 and P28, the normal adult pattern of supraspinal pain control fails to mature.

Our results point towards endogenous pain modulatory networks having a key role in the maturation of descending control over spinal nociceptive pathways. Microinjection of μ-opioid receptor agonists into the RVM resulted in dose-dependent descending facilitation in young rats, which switched to inhibition postnatal week 4. It is well established that RVM opioid signaling has a key role in descending pain control; RVM neurons express μ-, κ-, and δ-opioid receptors, and their activation produces variable degrees of analgesia [1,20,21]. Because ON cells are the only physiologically defined subpopulation of RVM cells to directly respond to μ-opioid receptor agonists [13,20], we can speculate that it is this group of cells that undergo key developmental changes in μ opioid signalling; however, this requires more evidence. We show that the consequences of activating RVM μ-opioid receptors changes dramatically during the late postnatal period. The fact that DAMGO mediates its effects over the same dose range at P21 and P40 suggests that the binding affinities of the μ-opioid receptor do not alter with age. The differences that we have seen cannot be attributed to diffusion of DAMGO to extra-NRM regions in younger brains, as comparisons of the preadolescent and adult brain show that brains from these 2 age-groups are of similar size [18]. Our results show that the effects of the delta agonist, deltorphin, in the RVM are not developmentally regulated, suggesting that we have identified a selective change in μ-opioid receptor-mediated activity. This is reinforced by the finding that GABA-mediated RVM inhibition is also unchanged, showing that the switch in μ-opioid receptor-mediated activity is not the result of an alteration in RVM GABA signalling. The switch in opioid-mediated activity in the RVM may reflect developmental regulation of the underlying pharmacology of this receptor at these different ages, but little is known about this in the RVM. Recently, changes in brainstem μ-opioid receptor molecular weight have been reported during development, which may have an impact on the binding properties of the receptor; however, the functional implications of this are, as yet, unclear [24]. In light of the data presented here, it is important that future efforts be made to investigate the ability of the μ-opioid receptor to bind agonists and antagonists, and to study whether the receptor is inserted and internalised from the cell membrane in the same way at all ages.

The fundamentally different functional consequences of RVM μ-opioid receptor activation at P21 and P40 is shown by the effects of μ-opioid receptor antagonism with CTOP, which inhibited nociceptive activity at P21 but had no effect at P40. Evidently, tonic ongoing activation of μ-opioid receptors by endogenous opioids occurs in the young RVM that is not present in adulthood. The postnatal period in both humans and animals is characterised by significantly lower mechanical and thermal pain thresholds, as well as by exaggerated and inappropriate responses to noxious stimuli [14]. This increased excitability has long been considered to be shaped by a lack of inhibitory neurotransmission within the spinal dorsal horn [2], much of which is intrinsic from GABA- and glycinergic spinal interneuronal populations. The data presented here highlight the important role of descending pathways in postnatal development; the presence of a tonic facilitation arising from endogenous opioid activation of RVM circuits in young animals is likely to make a major contribution to the excitability of spinal nociceptive circuits in young animals [15,18]. Nociceptive reflex and dorsal horn cell-receptive fields in young rats have low mechanical thresholds, large response amplitudes, and durations, and mature to the adult form only after P21 [14]. One might speculate that this enhanced excitability maximizes sensory input to the CNS, thus contributing to the activity-dependent tuning of spinal and supraspinal nociceptive circuits [7,30].

It is well known that neuronal circuits in the brain are shaped by experience during ‘critical periods’ in postnatal life. Activity-dependent development can be triggered by a variety of stimuli, depending on the brain region. Here we show, for the first time, that the development of brainstem descending pain pathways has a critical period in postnatal week 4, relatively late in postnatal life. Importantly, this is a period during which opioid signalling is required for the shift from descending facilitation to inhibition to take place. Discovery of this critical period helps to explain how early exposure to pain and injury may have long-term effects on sensory processing in later life. Neonatal injury and stress influences future pain processing both at the site of injury and globally, across the whole body [31,32]. Noxious stimuli cause release of endogenous opioids in a number of CNS regions [3,40]. Such a change in opioid signaling in the RVM around the critical period of postnatal development could permanently alter CNS pain processing by altering the normal balance of descending control over spinal pain networks. In support of this proposal, chronic administration of morphine over the second postnatal week accelerated the maturation of RVM descending controls. The recent report that neonatal injury can significantly alter opioidergic tone in adult PAG further supports a central role of opioids in setting the level of descending control of pain [25].

The tonic facilitation of nociceptive pathways by endogenous opiates in preadolescent rats might appear paradoxical, as morphine is an effective analgesic in young animals [36], but this is not so. Systemic morphine acts at many μ-receptor sites in the nervous system, at the level of the spinal cord, on C-fibre afferent terminals, and on numerous sites in the CNS, including the periaqueductal grey and other brainstem nuclei, mediated by distinct mechanisms [41]. Lesion experiments have emphasised the difference between sites of action for endogenous opioids and for systemic morphine [42]. Supplementary Fig. 2 shows that naloxone hydrochloride is able to reverse the increase in threshold elicited by systemic morphine in juvenile rats, and these data also suggest that peripherally restricted naloxone methiodide is also able to do the same. However, the naloxone methiodide data are not significantly different from any of the other groups in this study, implicating central rather than peripheral opioid receptors in morphine anti-nociception in juvenile rats.

We have shown that the preadolescent rat has an endogenous μ-opioid-mediated descending pain control system, which is tonically active and which facilitates rather than suppresses pain transmission. As the system matures, so the effects of activating this pain control system become increasingly inhibitory, so that excitation and inhibition are more finely balanced. We have also shown that the emergence of this balanced pain control is dependent on normal levels of activity in CNS opioid networks over a critical preadolescent period. This provides an explanation for individual differences in pain susceptibility based upon early life experience, whereby alterations in endogenous opioid levels at a critical stage of preadolescent life can shape top-down pain control in adult life.

## Conflicts of interest statement

The authors declare that they have no conflicts of interest with regard to this manuscript.
